# Ictal Electroencephalogram Visual Pattern Recognition of Seizure Adequacy During Electroconvulsive Therapy Treatment: A Step-by-Step Approach

**DOI:** 10.21315/mjms2023.30.2.7

**Published:** 2023-04-18

**Authors:** Kenny Ong Kheng Yee, Chee Kok Yoon, Zamtira Seman, Chhoa Keng Hong, Siti Nor Fadhlina Misron, Chin Han Lim

**Affiliations:** 1 NEURON (Neuropsychiatry and Neuromodulation Unit), Department of Psychiatry and Mental Health, Kuala Lumpur General Hospital, Kuala Lumpur, Malaysia; 2 Sector for Biostatistics and Data Repository, National Institutes of Health, Selangor, Malaysia; 3 General Psychiatry Unit, Hospital Permai, Johor, Malaysia

**Keywords:** electroconvulsive therapy, electroencephalogram, inter-rater reliability, seizure adequacy, visual pattern recognition

## Abstract

**Background:**

The NEURON (Neuropsychiatry and Neuromodulation Unit) electroconvulsive therapy electroencephalogram (ECT-EEG) Algorithmic Rating Scale (NEARS) is a step-by-step approach to ictal electroencephalogram visual pattern recognition of seizure adequacy based on recruitment, amplitude, symmetry, duration and degree of post-ictal suppression. The objectives of this clinical audit were to determine the degree of agreement on the NEARS operational criteria between two neuropsychiatrists, the reliability of electroconvulsive therapy practitioners’ administration of NEARS during ECT procedures and the correlation of NEARS scores with Clinical Global Impression scale scores after each ECT treatment session.

**Methods:**

Systematic random sampling was conducted. Even numbers of ictal tracings were selected for analysis from the total samples collected over 8 consecutive days of ECT overseen by a total of eight different ECT practitioners. Cohen’s kappa coefficient was used to measure the inter-rater reliability of the two neuropsychiatrists and determine the level of agreement between NEARS scores and those of the ECT practitioners. The correlation using NEARS scores and post-ECT Clinical Global Impression scores was measured with Spearman’s test. The significance level was set at *P* < 0.05.

**Results:**

Cohen’s kappa showed perfect agreement between the two neuropsychiatrists, at κ = 1.00 (SE = 0.001; *P* < 0.001), and strong agreement between NEARS scores of overall seizure adequacy and the scores interpreted by the ECT practitioners, at κ = 0.83 (95% CI: 0.66, 0.99; *P* < 0.001). Spearman’s test showed a weak negative association between NEARS scores and post-ECT Clinical Global Impression scores (*r* = −0.018; *P* = 0.900).

**Conclusion:**

NEARS may facilitate a brief, objectively reliable and practical assessment of ictal electroencephalogram quality. The scale is readily applicable by any trained ECT practitioner during an ongoing ECT procedure, especially when a prompt treatment decision is required.

## Introduction

Electroconvulsive therapy (ECT) is a safe and effective treatment modality that has been frequently utilised for the acute relief of certain patients’ psychiatric illnesses and their detrimental sequelae. In such therapy, rapid therapeutic responses are required with no or minimal adverse effects. During the ECT treatment session, when a seizure has been induced, the subsequent crucial step will be to determine the adequacy of the seizure based on an ictal electroencephalogram (EEG) recording, as this informs the decision about whether to further increase the stimulus dose or stop the session for the day.

The ECT-EEG is structured into the recruitment, polyspike, polyspike and slow wave, termination and post-ictal suppression phases. The duration, amplitude and morphology of each of these peri-ictal phases may differ inter-individually as well as intra-individually when seizure is adequately induced. Seizure duration, whether motor or ictal, has been shown to be an inadequate parameter for ascertaining ictal EEG quality, as other potential markers need to be evaluated (1, 2). Post-ictal suppression appears to be the ictal index most frequently associated with superior therapeutic outcomes (3, 4). Other indices include recruitment phase duration (5), early and mid-ictal amplitude (6, 7), and symmetry or interhemispheric coherence (8, 9).

The measures for ictal EEG interpretation can be generally categorised into quantitative (such as computer-generated EEG data, spectral analysis, the largest Lyapunov exponent and fractal dimensions) and manually rated measures (10). However, a gold standard measurement of seizure adequacy in ECT has yet to be established. In particular, a rating scale is needed that assists the ECT practitioner in making a brief and reliable assessment of ictal EEG quality. The NEURON (Neuropsychiatry and Neuromodulation Unit) ECT-EEG Algorithmic Rating Scale (NEARS) was therefore developed to evaluate seizure adequacy based on the ictal EEG recording during an ongoing ECT procedure. As the scale was recently constructed, a clinical audit of NEARS was conducted with the objectives of determining: i) the degree of agreement on the NEARS operational criteria between two neuropsychiatrists who were qualified and experienced in administering and training for ECT; ii) the reliability of ECT practitioners in the administration of NEARS during ECT procedures, where NEARS was assigned as the standard, and agreement among the ECT practitioners was compared with the standard; and iii) the correlation of NEARS scores with Clinical Global Impression (CGI) scale scores as the clinical outcome after each ECT treatment session.

## Methods

### NEARS Interpretation

NEARS is a step-by-step approach to ECT-EEG or ictal EEG visual pattern recognition of seizure adequacy based on a two-channel EEG recording after administration of a stimulus dose during the ECT procedure. NEARS was constructed by two neuropsychiatrists as part of this study. Prior to utilising the NEARS operational criteria, the calibration of the ECT-EEG tracing was set at 0.020 mV/mm or 200 uV/cm, depending on the ECT machine type. All ECT-EEG tracings were recorded via bifrontopolar-mastoid electrode placement. The sequential visual analysis of the ictal EEG was based on five indices as follows:

- Recruitment: the recruitment phase immediately post-stimulus, with a low-amplitude and high-frequency waveform (alpha or beta waves) and a duration of not more than 5 s prior to the appearance of the hypersynchronous polyspike phase
- Amplitude: a bilateral EEG amplitude of at least 1.5 cm (15 mm) in height from the peak to the trough of the amplitude in slow-wave complexes (overall maximum amplitude voltage in delta waves), with a total duration of at least 10 s
- Symmetry: interhemispheric symmetry in the ictal EEG (at least 50% of the time) from the start of the recruitment phase to the end of the slow-wave phase
- Duration: EEG seizure duration of at least 15 s, from the start of the recruitment phase to the end of the termination phase
- Degree of post-ictal suppression: abrupt termination endpoint or abrupt flattening of EEG immediately post-seizure termination, as measured by an automated adequacy of at least 50% or post-ictal suppression index (PSI) of at least 80% (the use of adequacy or PSI depends on the ECT machine model).

After assessing the ictal EEG indices, the interpretation of NEARS is then based on overall seizure adequacy as measured by the five indices. The seizures induced by ECT are categorised as adequate, equivocal or inadequate depending on the number of indices: four or five out of the total five indices are adequate markers of ictal EEG quality, three indices are equivocal and zero to two indices are indicative of an inadequately induced seizure.

However, there are some caveats with NEARS: i) in the elderly population, ECT-EEG tracing may appear less symmetrical, with lower amplitude, poor post-ictal suppression and shorter duration; ECT-EEG seems to be sub-optimal; ii) in bilateral ECT, higher amplitudes, more pronounced symmetry and post-ictal suppression may be observed compared to unilateral ECT; and iii) although bilateral EEG seizure duration is of at least 15 s, the termination phase may be prolonged, with poor regularity and low amplitude, i.e. a disorganised pattern with diffuse slowing.

### Electroconvulsive Therapy Administration

The study was conducted at a large tertiary hospital in the central region of Malaysia, where an average of 10 inpatients were administered ECT on Mondays, Wednesdays and Fridays. These cases include those on acute courses of six to eight treatment sessions over periods of 2 weeks to 3 weeks, as well as those on maintenance ECT (with treatment intervals ranging from two to four weekly). All procedures were administered in an ECT suite by an ECT practitioner facilitated by ECT co-ordinators and an anaesthesia provider. Only one ECT practitioner (a trained and privileged medical officer) was scheduled for each day of ECT on a rotational basis. All ECT was performed with a MECTA spECTrum 5000M machine (Mecta; Portland, OR, USA) at a pulse width of 1.00 ms and a constant current of 800 mA. EEGs were recorded with bifrontopolar-mastoid placement, i.e. the centre of the frontopolar electrode was positioned at 1.5 cm above the midpoint of each eyebrow, with the mastoid electrode placed on the mastoid bony prominence. All patients were prescribed bilateral ECT (bifrontotemporal electrode placement), with stimulus doses administered according to the titration dose method (11). Propofol was administered as the induction agent for all cases, with succinylcholine as the depolarising muscle relaxant. The modified CGI scale was utilised to assess the severity of psychiatric illnesses before and after each ECT procedure. CGI was rated on a 7-point scale, with a range of responses from 0 (normal, not at all ill) to 6 (the most unwell) (12).

### Study Procedure

Initially, ECT-EEG recording samples (generated by the ECT machine) were randomly chosen and analysed by the two neuropsychiatrists based on NEARS operational criteria to determine the degree of agreement. Subsequently, a clinical audit was performed on the ECT practitioners’ use of NEARS to evaluate the adequacy of ECT-EEG tracings on treatment days. Prior to the audit, the ECT practitioners were required to be trained in the application of NEARS to standardise its interpretation, reduce the extent of variability and identify any potential sources of error (e.g. their ability to identify common artefacts, such as movement and muscle artefacts).

Systematic random sampling was conducted. Even-numbered ECT-EEG tracings were selected from the total samples collected over 8 consecutive days of ECT days overseen by a total of eight different ECT practitioners. Any tracings with excessive artefact interference or ambiguous recording were discarded (of which there were three due to movement artefacts in the post-ictal phases). The ictal EEG tracings included the cases on acute courses and maintenance therapy, and from any treatment sessions.

### Statistical Analysis

Descriptive statistics were employed to describe the characteristic profiles of the ECT practitioners and patient sample. The calculated sample size for this study was 49 ECT-EEG tracings based on kappa coefficient κ1 and κ2 values of 0.5 and 0.8, respectively, with the power set at 90.0% and a significance level of 5% (13). Cohen’s kappa coefficient was used to measure inter-rater reliability between the two neuropsychiatrists for objective i) based on overall seizure adequacy (adequate, equivocal or inadequate) and objective ii) to determine the level of agreement between NEARS scores as determined by one of the neuropsychiatrists and by the ECT practitioners. Spearman’s correlation test was administered to determine the correlation between NEARS scores (on overall seizure adequacy) and post-ECT CGI scores. All analyses were performed using SPSS software version 26.0 (IBM Corp., 2019). The significance level was set at a *P*-value of less than 0.05.

## Results

In this study, 28 patients were recruited from the selected sample size of 49 ECT-EEG tracings. The characteristic ECT profiles of the 28 patients are presented in Table 1. Majority of the patients were female (71.4%), with an average age of 41.9 (SD = 16.19) years old. In terms of psychiatric diagnosis, schizophrenia accounted for the highest percentage, with 57.1%, and most of the patients (85.7%) were on a combination of at least two medications (antipsychotics, antidepressants, mood stabilisers or benzodiazepines). With regards to ECT, 71.4% of the patients were in the acute phase, predominantly for rapid or definitive responses due to the severity of their psychiatric illness (67.9%). The average number of treatment sessions was 3.4 (SD = 2.69) for patients on acute ECT, and the treatment interval for those on maintenance ECT was 22.9 (SD = 15.93) days. The mean stimulus dose (in millicoulombs) was 450.12 (SD = 427.34).

Table 2 shows that most of the ECT practitioners (six out of eight) were not trainees in a post-graduate programme in psychiatry. These practitioners had diverse working experience in a psychiatry department and ECT administration, ranging from 15 months–72 months and 12 months–70 months, respectively.

Cohen’s kappa showed a perfect agreement between the two neuropsychiatrists, at κ = 1.00 (SE = 0.001; *P* < 0.001), and a strong agreement between the NEARS scores of overall seizure adequacy and the scores interpreted by the ECT practitioners, at κ = 0.83 (95% CI: 0.66, 0.99; *P* < 0.001). Based on Spearman’s correlation test, there was a weak negative association between NEARS scores (of overall seizure adequacy) and post-ECT CGI scores (*r* = −0.018; *P* = 0.900).

## Discussion

NEARS facilitates a brief but objectively reliable and practical assessment of ictal EEG quality prior to the decision of whether to adjust the stimulus dose, conclude the treatment session for that day or switch to alternative electrode placement for subsequent sessions. The scale is readily applicable by any trained ECT practitioner during an ongoing ECT procedure, especially when a prompt treatment decision is required in a busy setting with a high patient load. Furthermore, as ECT is an aerosol-generating procedure with a high risk of pathogenic transmission, precautionary measures need to be undertaken to minimise the time spent on the procedure (especially in a centre with a relatively small treatment area, where physical distancing may be difficult) while simultaneously ensuring the therapeutic effectiveness of ECT.

The interpretation of ictal tracings during the ECT procedure may be obscured by the presence of artefacts, especially due to accidental dislodging of the recording electrodes, the manipulation of the patient’s head by the anaesthesia provider or improper placement of the recording electrodes prior to ictal onset. However, such tracings with gross artefacts were detected and not included in the analysis. Despite the artefacts’ appearance in a real clinical setting, the ability to correctly read the ECT-EEG tracings based on the operational criteria is invariably integral in ECT procedures. Whether the tracings are from the same or different patients, it is important for ECT practitioners to be aware that the determinants of the seizure threshold (and thus the tracing quality) in each patient are multifactorial and may change throughout the course of ECT—for example, the use of concurrent medication regimes that may differ from one treatment session to another, changes in the treatment session interval, any electrolyte imbalance or changes in oxygen or carbon dioxide blood levels (14–17).

The ECT practitioners in this study incorporated only medical officers from a tertiary hospital. Thus, the results may not be generalisable to other groups of practitioners. However, these medical officers were trained as a group by the two neuropsychiatrists in the use of NEARS prior to the study process, with pre-test and post-test assessments. Although the characteristic profiles of the ECT practitioners showed that two out of eight were trainees in a post-graduate programme in psychiatry, proper training on using the scale is of paramount importance so that any non-trainee medical officers with any degree of experience in ECT administration are equipped with the necessary skills and knowledge prior to implementing NEARS. A Cohen’s kappa level of agreement of 0.83 is strongly acceptable in this study between the NEARS operational criteria and the ratings of the ECT practitioners (18). The generalisability of the results was limited by the use of exclusively bifrontotemporal electrode placement and propofol as an induction agent and the fact that most of the ECT-EEG tracings were from cases with a schizophrenia diagnosis.

In this study, with the increase in NEARS scores, i.e. more adequately induced seizures, the corresponding post-ECT CGI scores decreased, indicating less severe psychiatric illness. Although the association was weak and not statistically significant, the purpose of NEARS was to ensure that each treatment session was administered in an optimal yet effective manner while minimising any potential adverse effects due to inadvertent excessive dose stimulation. The limitation of non-standardised interpretations of the CGI could have resulted in the post-ECT CGI scores being inconsistent and insignificant. Further research on the clinical utility of NEARS and its validity as a predictor of clinical response may need to examine the potential relationship between its operational criteria and the determinants of seizure threshold (e.g. electrode placement or pulse width) as well as the clinical outcomes with psychiatric illnesses using more specific rating scales.

## Figures and Tables

**Table 1:** Characteristic profile on 28 ECT patients (with 49 ECT-EEG tracings)

Characteristics	Frequency	%
Gender		
Male	8	28.6
Female	20	71.4
Psychiatric diagnosis		
Schizophrenia	16	57.1
Depressive Disorder	3	10.7
Bipolar Mood Disorder	6	21.4
Schizoaffective Disorder	2	7.1
Autism Spectrum Disorder	1	3.6
Concurrent psychotropic medication		
Antipsychotic	3	10.7
Antidepressant	1	3.6
Combination of at least two medications (antipsychotic/antidepressant/mood stabiliser/benzodiazepine)	24	85.7
ECT schedule		
Acute	20	71.4
Maintenance	8	28.6
Indication for ECT		
Rapid or definitive response required	19	67.9
Previous good response to ECT	8	28.6
Actively suicidal or life-threatening situation	1	3.6

**Characteristics**	**Mean**	**SD**

Age (years old)	41.9	16.19
Treatment session number (for acute ECT)	3.4	2.69
Treatment interval in days (for mECT)	22.9	15.93
Stimulus dose (mC)	450.12	427.34

Notes: ECT = electroconvulsive therapy; mECT = maintenance ECT; mC = millicoulomb; SD = standard deviation

**Table 2:** Characteristic profile on ECT practitioners

ECT day	Experience working in a psychiatry department (in months)	Experience on ECT administration (in months)	Status as a trainee in a post-graduate programme in psychiatry (in months)
1st	72	69	Non-trainee
2nd	72	53	48
3rd	62	36	Non-trainee
4th	22	22	Non-trainee
5th	50	44	Non-trainee
6th	72	70	36
7th	68	65	Non-trainee
8th	15	12	Non-trainee

Note: ECT = electroconvulsive therapy
